# Application of Syndromic Panels for respiratory Tract Infections in Lung Transplantation: A Critical Review on Current Evidence and Future Perspectives

**DOI:** 10.1111/tid.14448

**Published:** 2025-01-30

**Authors:** Andrea Lombardi, Giulia Renisi, Arianna Liparoti, Chiara Bobbio, Alessandra Bandera

**Affiliations:** ^1^ Infectious Diseases Unit Foundation IRCCS Ca' Granda Ospedale Maggiore Policlinico Milan Italy; ^2^ Department of Pathophysiology and Transplantation University of Milan Milan Italy

**Keywords:** fast microbiology, lung transplant, multi‐drug resistance, prophylaxis, syndromic panel

## Abstract

**Background:**

Infections are a common complication among lung transplant recipients (LuTR), particularly in the first year post‐transplant, with respiratory tract infections (RTI) being predominant. Syndromic molecular panels have been suggested to reduce morbidity and mortality by providing a diagnosis quickly. However, integrating these panels into clinical practice remains debated.

**Methods:**

An electronic search was conducted in PubMed, Scopus, and Embase to identify studies on applying syndromic panels for RTI diagnosis in LuTR. Three reviewers independently screened‐extracted data from relevant studies, focusing on concordance between syndromic panels and standard microbiologic tests and reporting isolates not detected by syndromic panels.

**Results:**

Four studies met the inclusion criteria, including 308 patients. The BioFire FilmArray Pneumonia Panel was the syndromic panel most frequently employed. In three studies, the syndromic panel was employed in LuTR with suspected RTI or during routine surveillance bronchoalveolar lavage; only in one case was the syndromic panel employed during the transplant procedure on samples from the donor. Agreement between syndromic panels and standard tests ranged from 0.56 to 0.98, with result turnaround times between 2.3 and 21.2 h. Sensitivity ranged from 58% to 94%, and specificity from 78% to 100%. Pathogens outside syndromic panels targets but identified by standard tests included *Candida* spp., unspecified gram‐negative rods, and *Aspergillus* spp.

**Conclusion:**

Syndromic panels offer a faster alternative to standard microbiologic tests. However, they miss numerous possible pathogens, highlighting the necessity for concurrent standard testing. Further research is needed to establish the most efficient integration of syndromic panels in LuTx infection diagnostic.

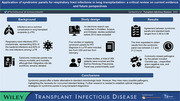

## Graphical abstract

Limited data on syndromic panels for detecting respiratory tract infections in lung transplant candidates and recipients are available. While these panels provide a rapid turnaround time, they may fail to identify specific pathogens relevant in this setting, such as fungi.

**Abbreviations**
BAL: bronchoalveolar lavage
LuTx: lung transplant
PCR: polymerase chain reaction
RTI: respiratory tract infections
LuTR: lung transplant recipients
SOC: standard of care

## 1 Introduction

Infections in lung transplant (LuTx) recipients (LuTR) are a frequent event, especially in the first year after transplant. It has been estimated that infections, excluding cytomegalovirus, are responsible for about 35% of deceases in the first 12 months after transplant, representing the first cause of death overall [1]. Respiratory tract infections (RTI) predominate, representing almost 50% of the bacterial infections and 80% of the viral infections reported among LuTR in the Swiss Transplant Cohort [2].

Syndromic molecular panels screen for predefined targets associated with a specific syndrome, most commonly for respiratory, gastrointestinal, neurological, or sexually transmitted disease presentation. Multiplex panels, which allow for the simultaneous detection of multiple targets (usually more than five pathogens), have been advocated to curb morbidity and mortality among patients with ongoing infections. They potentially allow the rapid identification of the culprit pathogen(s) or the presence of a peculiar resistance mechanism(s), thus the prompt introduction of targeted treatments [3]. These diagnostic tests are not without limitations. They are often associated with high costs, and their heightened sensitivity can pose challenges for clinicians in interpreting the clinical significance of the detected organisms and/or resistance genes, particularly when multiple targets are identified simultaneously. This can lead to inappropriate treatment and unnecessary subsequent laboratory testing. Furthermore, the optimal integration of syndromic panels into laboratory workflows, and the monitoring of their accuracy, remain subject to ongoing debate [4].

In transplantation, evidence‐based recommendations are lacking to inform the optimal use of syndromic panels. Standard diagnostic testing methods continue to play a central role in the diagnosis of infection. However, these assays are often limited by suboptimal sensitivity and/or prolonged turnaround times, which contribute to missed and/or delayed diagnoses in solid organ transplant recipients, thus supporting the implementation of syndromic panel use [5].

Currently, there is no clear consensus on the significance of syndromic panel application in LuTx, and thus, this technology is implemented variably. To understand its use and the possible impacts in the field of LuTx, we reviewed the literature describing the employment of syndromic panels to assess RTI among donors and recipients.

## 2 Methods

### 2.1 Data Sources and Search Strategies

The electronic search was performed on PubMed, Scopus, and Embase databases on 10/06/2024. There was no restriction on language. The search strategy was designed and conducted by an experienced researcher with inputs from the other study's investigators. Files S1 and S2 contain the search strategy for each database and the PRISMA flowchart of the study.

### 2.2 Type of Outcome Measure

The primary outcome was the concordance between the syndromic panel and the standard of care (SOC) microbiologic test. The secondary outcome was a description of isolates not detected by syndromic panels, including those not detectable because they were not included in the panel targets.

### 2.3 Study Selection and Data Extraction

We included individual studies that reported the application of syndromic panels in LuTR to diagnose RTI.

We excluded studies published before 2010 and included English, French, and Italian studies.

Three reviewers screened all titles and abstracts independently. Studies included at this level by either reviewer were included for full‐text screening by another reviewer, and disagreements were resolved through discussion with the study coordinator. We extracted data for each report in a specifically designed data collection form.

Extracted data included the test employed, the number of patients included, the material on which the test was performed, the results of the SOC microbiologic tests and those provided by the syndromic panel, the concordance between the results of the syndromic panel and SOC test, the time necessary to obtain results, sensitivity and specificity of the syndromic panel and the positive and negative predictive value of the syndromic panel.

No approval from the ethical committee was required due to the nature of the study.

## 3 Results

Four studies were included [6, 7, 8, 9], two from USA and two from Germany. The BioFire FilmArray Pneumonia Panel and its Plus version were employed in three studies, while the first syndromic panel reported in the literature in this setting was the Curetis P55 Pneumonia syndromic panel. SOC methods were reported as conventional cultures for bacteria and fungi and conventional viral testing; only in the study of Hoover et al. was the use of NxTag respiratory panel reported for viral detection. Table S1 reports the microorganisms and resistance genes detected by the syndromic panels included in the study.

A total of 308 patients were included in the four studies. In three studies, the syndromic panel was employed in LuTR with suspected LRTI or applied on samples collected during routine surveillance bronchoalveolar lavage (BAL); only in one case was the syndromic panel employed during the transplant procedure on samples from the donor.

The agreement between the syndromic panel and the SOC ranged from 0.56 to 0.98, while the time requested to obtain results from the syndromic panel was between 2.3 and 21.2 h. Based on the studies where the data was provided, sensitivity for syndromic panels ranged from 58% to 94%, whereas specificity ranged between 78% to 100%. Table 1 presents details of patients considered, samples tested and syndromic panel performance.

**TABLE 1:** Studies included details of patients considered, samples tested, and syndromic panel performance.

Study	Population type	Test employed	Sample analyzed	Patients, *n*	PCR+ samples, *n* (%)	SOC+ samples, *n* (%)	PCR‐SOC agreement	TTR PCR, h (IQR)	TTR SOC, h (IQR)	Sensitivity, % (CI)	Specificity, % (CI)	PPV, % (CI)	NPV, % (CI)
Drick, 2018[6]	LTx recipients with suspected LTRI	Curetis P55 Pneumonia	BAL fluid	48	20 (42)	32 (67)	0.56^a^	21.2 (19.3–65.7)	23 (21.1–67.4)	66^b^ (46.8–81.4)	100^b^ (79.4–100)	100^b^(84–100)	59^b^ (39–78)
Hoover, 2020[7]	LTx recipient (68% routine BAL)	BioFire FilmArray Pneumonia Panel	BAL fluid	150	23 (15)	22 (15)	0.98 (0.95–1)° bacteria	3.8 (2.8‐5.1)	48 (46–70) bacteria	94^b^	78^b^	97^b^	64^b^
					22 (15)^b^	18 (12)^b^	0.92 (0.87–0.97)° viruses		13 (10–34) viruses				
Kayser, 2021[9]	LTx recipient with suspected LTRI	BioFire FilmArray Pneumonia Panel Plus	BAL fluid	60	43 (72)	47 (78)	0.564^a^ ^b^ bacteria	2.3 (2‐2.8)	25.2 (22.8–69.5) bacteria	58^b^	100^b^	na	na
							0.693^a^ viruses		23.4 (21.1–62) viruses	na	na	100^b^	93^b^
Nguyen, 2023[8]	LTx donor at organ recovery	BioFire FilmArray Pneumonia Panel	BAL fluid	50	na	na	0.98 (0.97–0.99)°	2.4 (2‐6.4)	66.2 (47.2–86.5) bacteria; 4.6 (1.9–6) viruses	na	na	na	na
	Graft at implantation		Airway swab/bronchial tissue		na	na	0.98 (0.97–0.99)/0.97 (0.96–0.98)	na	100.4 (58.9–122.6)/165.2 (97–342.6)	na	na	na	na
	LTx recipient after Tx		BAL fluid		na	na	0.97 (0.96–0.98)	na	49.4 (46.8–74.2) bacteria; 16.4 (8.6–29.2) viruses	na	na	na	na

Abbreviations: BAL, bronchoalveolar lavage; IQR, interquartile range; na, not available; NPV, negative predictive value; PCR+, positive syndromic panel; PPV, positive predictive value; SOC, standard of care; SOC+, positive standard of care; TTR, time to results.

^a^ Cohen's kappa °Gwet's AC1.

^b^ Considering only targets detected by a syndromic panel.

**FIGURE 1:**
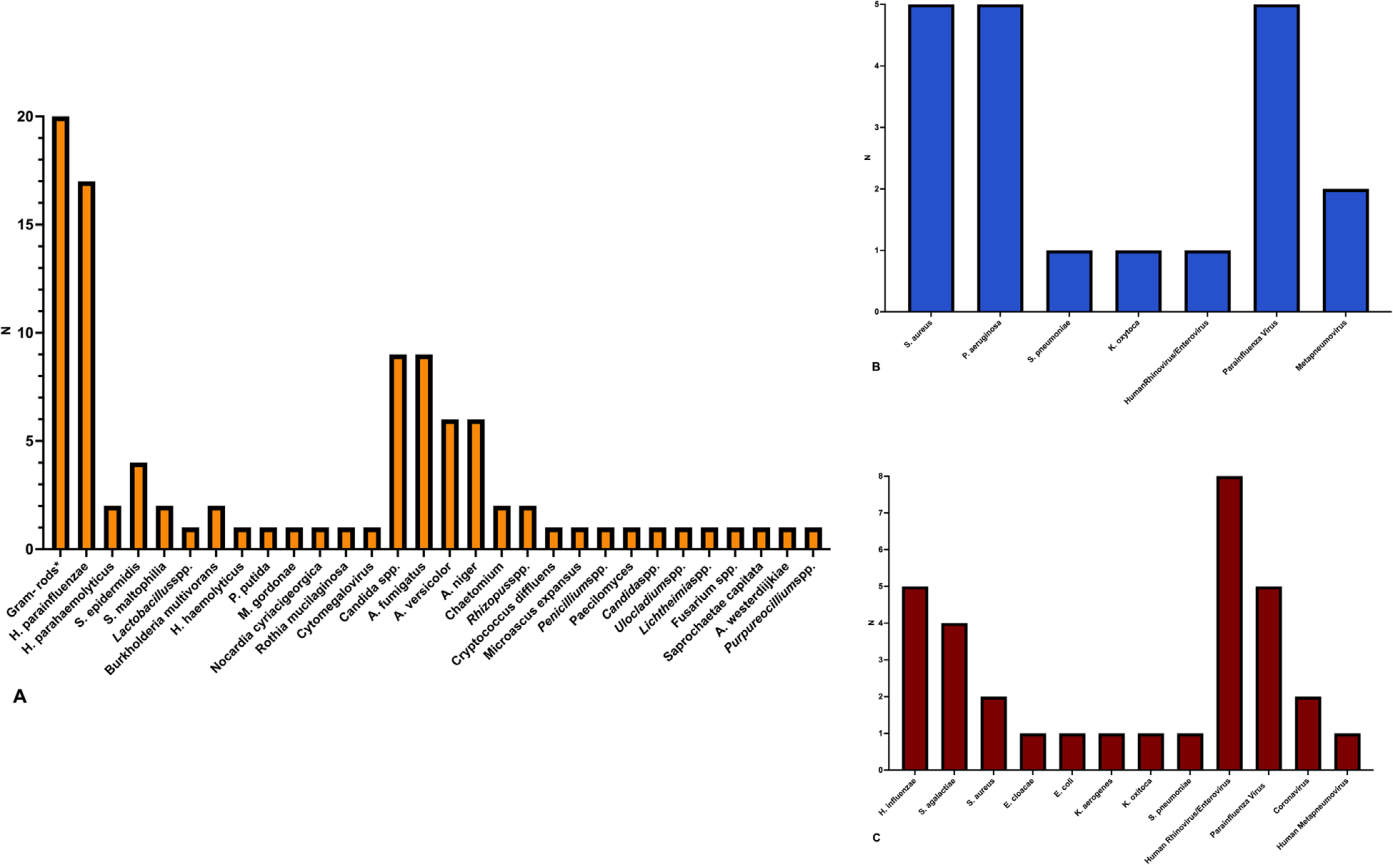
Frequencies of microorganisms not identified by syndromic panels due to being outside the technique's targets (Panel A), missed by syndromic panels despite being within the targets (Panel B), and identified exclusively by syndromic panels but missed by standard‐of‐care (SOC) methods (Panel C). (*Gram‐rods were reported by Hoover et al. and Nguyen et al. without further detail. °For Nguyen et al., only the syndromic panel and SOC results from donor bronchoalveolar lavage [BAL] were considered.).

The most frequently detected microorganisms not targeted by syndromic panels but identified through SOC methods were unspecified Gram‐negative rods, followed by *Haemophilus parainfluenzae*, *Candida* spp., and *Aspergillus* spp. (Figure 1, Panel A). Among the microorganisms targeted by syndromic panels but detected only by SOC, the most commonly identified were *Staphylococcus aureus*, *Pseudomonas aeruginosa*, and Parainfluenza virus (Figure 1, Panel B). It is important to note that the majority of missed relevant bacteria, specifically *S. aureus* and *P. aeruginosa* (four instances each), were reported in the study by Drick et al. This study utilized a different syndromic panel compared to the other studies, limiting the generalizability of these findings. Lastly, Human Rhinovirus/Enterovirus, Parainfluenza virus, and *H. influenzae* were the microorganisms most frequently identified exclusively by syndromic panels (Figure 1, Panel C).

## 4 Discussion

A scarce amount of data is available regarding the application of syndromic panels in detecting RTI among LUtx candidates or recipients. We have identified only four studies employing, in most cases, the BioFire FilmArray Pneumonia Panel, primarily in LuTR with suspected LTRI. Overall, the syndromic panels provided results in a far inferior time compared to SOC, with an agreement between the two approaches comprised between 0.56 and 0.98. Based on the studies that reported them, the sensitivity and specificity of syndromic panels ranged between 58%–94% and 78%–100%, respectively. Many microorganisms, especially unspecified gram‐rods, *H. parainfluenzae*, *Candida* spp., and *Aspergillus* spp., were not identified by the syndromic panels, mainly because those microbes were not among the targets included in the syndromic panel.

The application of syndromic panels in infectious diseases is a recent acquisition with a growing number of experiences. As shown by our work, the experience of syndromic panel application among LuTx candidates or recipients is limited. In a recently published trial, Markussen and colleagues highlighted how the use of a syndromic panel led to faster and more targeted antimicrobial treatment for patients with suspected community‐acquired pneumonia having access to the emergency department, with a hazard ratio for intervention in the syndromic panel group compared with the SOC group of 3.08 (95% confidence interval, 1.95–4.89) [10]. The result is interesting, but it was obtained in a population with very different characteristics related to the host and the possible culprit pathogens than the one composed by LuTR. In another publication from the same group, the syndromic panel increased the pathogen detection rate from 62.8% with SOC methods to 81.3%. Interestingly, the most frequent isolates were *H. influenzae*, severe acute respiratory syndrome coronavirus 2 (SARS‑CoV‑2), and *S. pneumoniae*, and the microorganisms most commonly not identified by the syndromic panel were SARS‐CoV‐2 and the respiratory syncytial virus [11]. While the first one was related to the coronavirus disease 2019 pandemic of the past years and has significantly reduced its relevance, the second one is a pathogen that could lead to severe clinical manifestations among immunocompromised hosts like LuTx recipients and thus should not be missed.

Our review shows that syndromic panels frequently miss fungi because these microorganisms are not included among the panel's targets. Syndromic panels are designed for use in the general population, where fungal infections are rare and not deemed a significant diagnostic priority. However, in LuTRs, fungi can cause severe and life‐threatening diseases, making their detection critical for initiating appropriate treatment. However, it is important to note that not all fungi identified in the studies should be considered true pathogens. For instance, *Candida* spp. may cause anastomosis infections and, in rare cases, pneumonia among LuTRs, but it is often a contaminant without substantial clinical significance. Additionally, fungal pathogens generally take longer than viruses or bacteria to cause significant clinical manifestations. As a result, the timing of antifungal therapy is less critical, and initiating treatment within a few hours of detection is usually unnecessary. SOC culture methods remain appropriate for detecting these potential pathogens, as they identify fungi and provide essential information on their susceptibility to antifungal agents—something syndromic panels cannot offer.

The conclusions regarding the agreement between syndromic panels and SOC methods are limited due to several factors: the small sample size, the lack of detailed information about the SOC techniques employed, and the use of two different syndromic panels across the four studies. The highest agreement was observed for viruses, which is unsurprising given that SOC methods for viral detection primarily rely on polymerase chain reaction—the same technology utilized by syndromic panels. For bacteria, however, significant variations were noted between studies, even when the same syndromic panel was used. A particularly notable finding is the considerable difference in the time required to obtain results between syndromic panels and SOC methods. While this difference is an expected outcome due to the intrinsic rapidity of syndromic panel technology, it highlights a key advantage: the potential for significantly reducing the time needed to implement therapeutic interventions.

Our study has some obvious significant limitations related to the scarce number of studies available on the topic. An overall minimal number of patients has been included in the studies, and there are differences related to the tests employed (Curetis P55 Pneumonia vs. BioFire FilmArray Pneumonia Panel vs. BioFire FilmArray Pneumonia Panel Plus) and the patient's condition on which the syndromic panel has been assessed (Recipient BAL in suspected LRTI vs. recipient surveillance BAL vs. donor BAL). Nonetheless, we believe that assessing the experiences available regarding the application of syndromic panels in diagnosing LTRI among LuTx donors and recipients is crucial to understanding in which direction future research efforts investigating the application of these tests should be made.

From these first experiences, it appears clear how applying a syndromic panel can help obtain etiological diagnosis faster than with the SOC methods, a crucial point to guarantee the best treatment and, at the same time, reduce the use of unnecessary antimicrobials. Moreover, the syndromic panels seem to have excellent sensitivity and specificity, thanks to the technology behind them. It is also clear that syndromic panels cannot identify all the culprit pathogens due to the limited number of targets owned, with many fungal and bacterial microorganisms going unnoticed. This is particularly crucial in LuTx, where rare and opportunistic pathogens are more prevalent than in the general population and associated with significant clinical manifestations. A potential application of syndromic panels in the field of LuTx lies within the transplant procedure itself. Syndromic panels could be utilized alongside SOC methods to analyze the donor's BAL, enabling rapid identification of bacterial and viral pathogens that the recipient's prophylaxis might not cover. Additionally, in the early post‐transplant period, syndromic panels could be applied to the recipient's BAL to confirm the absence of significant bacterial and viral pathogens. This would support the safe and timely discontinuation of prophylactic treatments, potentially reducing unnecessary exposure to antimicrobial agents.

Based on the current evidence, the use of syndromic panels can be considered in the field of LuTx, both on donors and recipients, aiming at obtaining faster etiological diagnosis that allows the quick inception of targeted therapies. Standard care technologies should always be performed concurrently to guarantee that no pathogens, especially fungi, are missed. Moreover, as stated by the syndromic panels' fact sheets, these techniques should be used in conjunction with culture to determine bacterial susceptibility or resistance [12]. Ideally, syndromic panel use among LuTx donors/recipients should be defined in specific algorithms, providing diagnostic and therapeutic indications according to the results obtained. Further research should be focused on this to find the right place for the syndromic panel in the context of the growing relevance of stewardship, both for diagnostic procedures and antimicrobials.

## Author Contributions

Andrea Lombardi, Giulia Renisi, and Alessandra Bandera conceived the study. Andrea Lombardi, Giulia Renisi, Arianna Liparoti, and Chiara Bobbio reviewed the literature. Andrea Lombardi wrote the first draft of the manuscript. All the authors reviewed the final version of the manuscript.

## Conflicts of Interest

The authors declare no conflicts of interest.

## Supporting information



Supporting Information

Supporting Information

Supporting Information

## Data Availability

The data that support the findings of this study are available upon request to the corresponding author.
