# Supplementary material for: Application of Syndromic Panels for respiratory Tract Infections in Lung Transplantation: A Critical Review on Current Evidence and Future Perspectives
Source: Transpl Infect Dis. 2025 Jan 30;28(1):e14448. doi: 10.1111/tid.14448 (PMC12892830; doi:10.1111/tid.14448)
Supplement: Supplementary file 1 — Supporting Information [file TID-28-e14448-s002.docx]

**Supplementary file 1.** Search strategy employed in each database.

**PubMed: 328 results**

("lung transplant*"[Title/Abstract] AND ("Pneumonia Panel Plus"[All Fields] OR "Biofire"[All Fields] OR "panel"[All Fields] OR "PCR"[All Fields] OR "Curetis"[All Fields])) AND ((english[Filter] OR french[Filter] OR italian[Filter]) AND (2010:2024[pdat]))

**Scopus: 256 results**

ABS((lung transplant*) AND (biofire OR panel OR pcr OR curetis)) AND PUBYEAR > 2019 AND PUBYEAR < 2025 AND ( LIMIT-TO ( DOCTYPE,"ar" ) ) AND ( LIMIT-TO ( LANGUAGE,"English" ) OR LIMIT-TO ( LANGUAGE,"French" ) OR LIMIT-TO ( LANGUAGE,"Italian" ) )

**Embase: 786 results**

'lung transplantation':ab AND ('pneumonia panel plus':ab OR 'pcr':ab OR 'curetis':ab OR 'panel':ab OR 'biofire':ab) AND ([english]/lim OR [french]/lim OR [italian]/lim) AND [2010-2024]/py
