# Supplementary material for: Application of Syndromic Panels for respiratory Tract Infections in Lung Transplantation: A Critical Review on Current Evidence and Future Perspectives
Source: Transpl Infect Dis. 2025 Jan 30;28(1):e14448. doi: 10.1111/tid.14448 (PMC12892830; doi:10.1111/tid.14448)
Supplement: Supplementary file 2 — Supporting Information [file TID-28-e14448-s001.docx]

**Identification of studies via databases and registers**

Records removed *before screening*:

Duplicate records removed (n = 823)

Records identified from:

PubMed (n = 328)

Scopus (n = 256)

Embase (n = 786)

**Identification**

Records screened

(n = 547)

Records excluded

(n = 234)

Reports sought for retrieval

(n = 313)

**Screening**

Reports excluded:

Not involving LuTx (n = 73)

Not employing syndromic panels (n = 196)

Other (n = 40)

Reports assessed for eligibility

(n = 313)

Studies included in review

(n = 4)

**Included**

This work is licensed under CC BY 4.0. To view a copy of this license, visit <https://creativecommons.org/licenses/by/4.0/>
