# Supplementary material for: Application of Syndromic Panels for respiratory Tract Infections in Lung Transplantation: A Critical Review on Current Evidence and Future Perspectives
Source: Transpl Infect Dis. 2025 Jan 30;28(1):e14448. doi: 10.1111/tid.14448 (PMC12892830; doi:10.1111/tid.14448)
Supplement: Supplementary file 3 — Supporting Information [file TID-28-e14448-s003.docx]

**Supplementary Table 1.** Microorganisms and resistance genes detected by the syndromic panels included in the study.

|  | **Curetis P55 Pneumonia PCR** | **BioFire Filmarray Pneumonia Panel** | **BioFire Filmarray Pneumonia Plus Panel** |
| --- | --- | --- | --- |
| **Gram-positive** | *Staphylococcus aureus* | *Staphylococcus aureus* | *Staphylococcus aureus* |
|  | *Streptococcus pneumoniae* | *Streptococcus pneumoniae* | *Streptococcus pneumoniae* |
|  |  | *Streptococcus pyogenes* | *Streptococcus pyogenes* |
|  |  | *Streptococcus agalactiae* | *Streptococcus agalactiae* |
|  |  |  |  |
| **Gram-negative** | *Citrobacter freundii* | *Escherichia coli* | *Escherichia coli* |
|  | *Escherichia coli* | *Enterobacter cloacae* | *Enterobacter cloacae* |
|  | *Enterobacter cloacae* complex | *Klebsiella aerogenes* | *Klebsiella aerogenes* |
|  | *Enterobacter aerogenes* | *Proteus* spp | *Proteus* spp |
|  | *Proteus species* | *Klebsiella pneumoniae* | *Klebsiella pneumoniae* group |
|  | *Klebsiella pneumoniae* | *Klebsiella oxytoca* | *Klebsiella oxytoca* |
|  | *Klebsiella oxytoca* | *Serratia marcescens* | *Serratia marcescens* |
|  | *Klebsiella variicola* | *Moraxella catarrhalis* | *Moraxella catarrhalis* |
|  | *Serratia marcescens* | *Pseudomonas aeruginosa* | *Pseudomonas aeruginosa* |
|  | *Moraxella catarrhalis* | *Acinetobacter* complex | *Acinetobacter calcoaceticus-baumannii* complex |
|  | *Pseudomonas aeruginosa* | *Legionella pneumophila* | *Legionella pneumophila* |
|  | *Acinetobacter baumannii* complex |  |  |
|  | *Stenotrophomonas maltophilia* |  |  |
|  | *Legionella pneumophila* |  |  |
|  |  |  |  |
| **Viruses** |  | Adenovirus | Adenovirus |
|  |  | Coronavirus | Coronavirus |
|  |  | Metapneumovirus | Metapneumovirus |
|  |  | Rhinovirus/Enterovirus | Rhinovirus/Enterovirus |
|  |  | Influenza A | Influenza A |
|  |  | Influenza B | Influenza B |
|  |  | Parainfluenza | Parainfluenza virus |
|  |  | RSV | RSV |
|  |  |  | MERS-CoV |
|  |  |  |  |
| **Others** | *Pneumocystis jirovecii* | *Haemophilus influenzae* | *Haemophilus influenzae* |
|  | *Haemophilus influenzae* | *Chlamydophila pneumoniae* | *Chlamydophila pneumoniae* |
|  | *Chlamydophila pneumoniae* | *Mycoplasma pneumoniae* | *Mycoplasma pneumoniae* |
|  | *Mycoplasma pneumoniae* |  |  |
|  |  |  |  |
| **Antibiotic resistance genes** |  | KPC, NDM, OXA-48 like, VIM, IMP | KPC, NDM, OXA-48 like, VIM, IMP |
|  |  | CTX-M | CTX-M |
|  |  | mecA/mecC and MREJ | mecA/mecC and MREJ |
| RSV, respiratory syncytial virus; MERS-CoV, Middle East Respiratory Syndrome Coronavirus; KPC, *Klebsiella pneumoniae* carbapenemase; NDM, New Delhi metallo-beta-lactamase; VIM, Verona integron-encoded metallo-beta-lactamase; IMP, imipenemase; MREJ, mec element right extremity junction. | | | |
